# Elements to assess the quality of information of case reports in pregnancy pharmacovigilance data—a ConcePTION project

**DOI:** 10.3389/fdsfr.2023.1187888

**Published:** 2023-06-29

**Authors:** Yrea R. J. van Rijt-Weetink, Khoezik Chamani, Antoine C. G. Egberts, Florence P. A. M. van Hunsel, David J. Lewis, Laura M. Yates, Ursula Winterfeld, Eugène P. van Puijenbroek

**Affiliations:** ^1^ Pharmacovigilance Centre Lareb, ’s-Hertogenbosch, Netherlands; ^2^ Groningen Research Institute of Pharmacy, PharmacoTherapy, -Epidemiology and -Economics, University of Groningen, Groningen, Netherlands; ^3^ Division of Pharmacoepidemiology and Clinical Pharmacology, Department of Pharmaceutical Sciences, Faculty of Science, Utrecht University, Utrecht, Netherlands; ^4^ Department of Clinical Pharmacy, University Medical Centre Utrecht, Utrecht, Netherlands; ^5^ Global Drug Development, Novartis Pharma GmbH, Wehr, Germany; ^6^ School of Life and Medical Sciences, University of Hertfordshire, Hatfield, England; ^7^ KRISP, University of Kwazulu-Natal, Durban, South Africa; ^8^ Northern Genetics Service, Newcastle-upon-Tyne, United Kingdom; ^9^ Swiss Teratogen Information Service and Clinical Pharmacology Service, Centre Hospitalier Universitaire Vaudois (CHUV) and University of Lausanne, Lausanne, Switzerland

**Keywords:** clinical quality, data quality, pregnancy, pharmacovigilance, teratology

## Abstract

To assess the risk of exposure to a medicinal product during pregnancy in an individual case report, the necessary information should be present, complete and clearly described. Previously designed grading tools were not developed for pregnancy pharmacovigilance data. This study aims to identify the elements that are necessary to assess of the quality of information for risk assessment of medicinal products used during pregnancy. This is a first step in the development of a validated method to assess the clinical quality of case reports in pregnancy pharmacovigilance data. Potential information elements were determined by means of an expert focus group discussion and a survey based on its outcome. This provided an overview of possible information elements to be selected. For the final selection of the elements, a second survey and subsequent focus group discussion was used. Twenty-one information elements within seven categories were identified: information related to the association itself, the event, exposure to the medicinal product, maternal factors, pregnancy, labour, and the child. This study identified elements considered necessary in the assessment of quality of information of case reports in pregnancy pharmacovigilance data, via an extensive four-step process.

## 1 Introduction

The majority of women use one or more medicinal products during their pregnancy. (Mitchell et al., 2011; Lupattelli et al., 2014). The effects of these treatments on mother and child are often unknown at the time of marketing authorization since pregnant women are usually excluded from clinical trials for ethical reasons. (Adam et al., 2011). Evidence and experience regarding adverse effects of medicinal products used during pregnancy has therefore to be obtained from daily life experiences after approval for marketing of the drug, i.e., by pharmacovigilance activities. Well known examples of risks that became apparent after products were marketed are thalidomide-induced phocomelia in the early sixties, adenocarcinoma of the vagina with diethylstilbestrol (DES), and more recently the neurodevelopmental effects after intrauterine exposure to valproate, or morphological changes associated with mycophenolate mofetil. (McBride, 1961; Herbst et al., 1971; Benevent et al., 2017; Perez-Aytes et al., 2017).

Several established approaches are currently used to capture and analyse real-world experiences and maternofoetal outcomes of medication use by pregnant women. (Benevent et al., 2017). Case reports documenting individual patient outcomes (adverse and normal) after medicinal products were used during pregnancy are an important source of information. Healthcare professionals or consumers can report such experiences to a national pharmacovigilance centre or the marketing authorisation holder (MAH) of the products. These spontaneous reports may be supplemented by solicited reports (e.g., from pregnancy registries, patient support programmes, pharmacoepidemiologic studies and non-interventional studies) all of which are assessed and analysed, which in turn may lead to the generation of safety signals. In addition to the aforementioned, MAHs are responsible for monitoring all reports of suspected adverse reactions (including congenital anomalies, and untoward occurrences during breastfeeding) and all exposures and outcomes to the use of their licenced products in pregnant women including reports published in the medical literature. (European Medicines Agency, 2017). Spontaneous and solicited reports and reports of cases of harm described in literature are forwarded in a structured format as Individual Case Safety Reports (ICSRs) to larger operating systems, such as EudraVigilance, operated by the European Medicines Agency (EMA), and VigiBase, maintained by the World Health Organisation (WHO) collaborating centre, the Uppsala Monitoring Centre (UMC). (Bergvall et al., 2014; European Medicines Agency, 2023). This pooling of data in the form of ICSRs is designed to facilitate the early detection and assessment of potential safety signals for medicinal products in general, but is currently used for capture of pregnancy exposure data too.

Another approach for gathering and analysing pregnancy data is via Teratology Information Services (TIS). (ENTIS, 2023; OTIS, 2023). TIS centres counsel healthcare professionals and sometimes consumers regarding exposure to medicinal products during pregnancy and lactation. Additionally, they may collect information on the pregnancy outcomes in cases where women have been exposed to medicinal products during pregnancy. TIS centres collaborate through the European Network of Teratology Information Services (ENTIS) and the Organization of Teratology Information Specialists (OTIS) to improve knowledge on the safety of medicinal product use during pregnancy and lactation.

Finally, pregnancy registries aim to follow women during pregnancy and lactation to collect information on possible exposures to medicinal products, pregnancy complications and outcomes (Benevent et al., 2017; Vorstenbosch et al., 2019). A similar methodology is used in MAH-initiated enhanced pharmacovigilance programmes, where spontaneously reported pregnancies are followed, using amongst others structured follow-up data collection at set intervals. (Geissbühler et al., 2020).

These various data collection methodologies each have their own goals, data elements and characteristics, which hampers exchange and interpretation of information. Information that is provided may vary among data sources (e.g., national pharmacovigilance centres, MAHs, reports from literature), type of reporters (e.g., healthcare professionals or consumers), and reported adverse outcomes, influencing the utility of the reports for assessing the safety of the medicinal products studied.

In order to reliably assess the likelihood of a causal association between an exposure and clinical outcome following exposure to a medicinal product during pregnancy in an individual case report, the necessary information should be present, complete and clearly described. High data quality at individual case report level is also key to enabling subsequent database analysis for safety signals. (Meyboom et al., 1997; Oosterhuis et al., 2018). Tools have previously been designed for the purpose of assessing either the completeness of pharmacovigilance data (VigiGrade, developed by the UMC), or the clinical quality of the information provided (ClinDoc, developed by pharmacovigilance centre Lareb). (Bergvall et al., 2014; Oosterhuis et al., 2018). These tools have specifically been designed for ICSRs regarding potential medicinal product exposure-adverse event associations in non-pregnant individuals, where the adverse event occurs in the individual using the product. However, the risk assessment regarding medicinal product exposure during pregnancy requires substantially different clinical information compared to non-pregnancy related assessments. For example, the latency of an adverse event is important in non-pregnancy causality assessments, while in pregnancy it is more important to be able to link the timing of exposure to the chronology of gestation. Additionally, the types of adverse events that can occur in a foetus are wide ranging and may differ depending on the gestational stage at exposure, and may only become apparent after birth. Linking mother and child can present a further challenge. Neither of the previously mentioned grading tools were developed specifically for monitoring drug safety during pregnancy.

Moreover, which elements of information are relevant may vary depending on the different situations in which the medicinal products are used, the nature of the effects and the gestational stage at exposure. The completeness of relevant information elements collected for pregnancy pharmacovigilance, and whether the collected information is suitable for a reliable risk assessment is currently unknown.

Therefore, the aim of this study was to identify elements that are necessary for the assessment of the quality of information for risk assessment of medicinal products used during pregnancy, as a first step in the development of a validated method to assess the clinical quality of case reports in pregnancy pharmacovigilance data. This study is part of Work Package 2 of the IMI funded ConcePTION project, in which national pharmacovigilance centres, MAHs and TIS centres collaborated in optimizing the collection, analysis and interpretation of reported pregnancy pharmacovigilance data. (ConcePTION, 2019).

## 2 Methods

### 2.1 Setting and design

**FIGURE 1:**
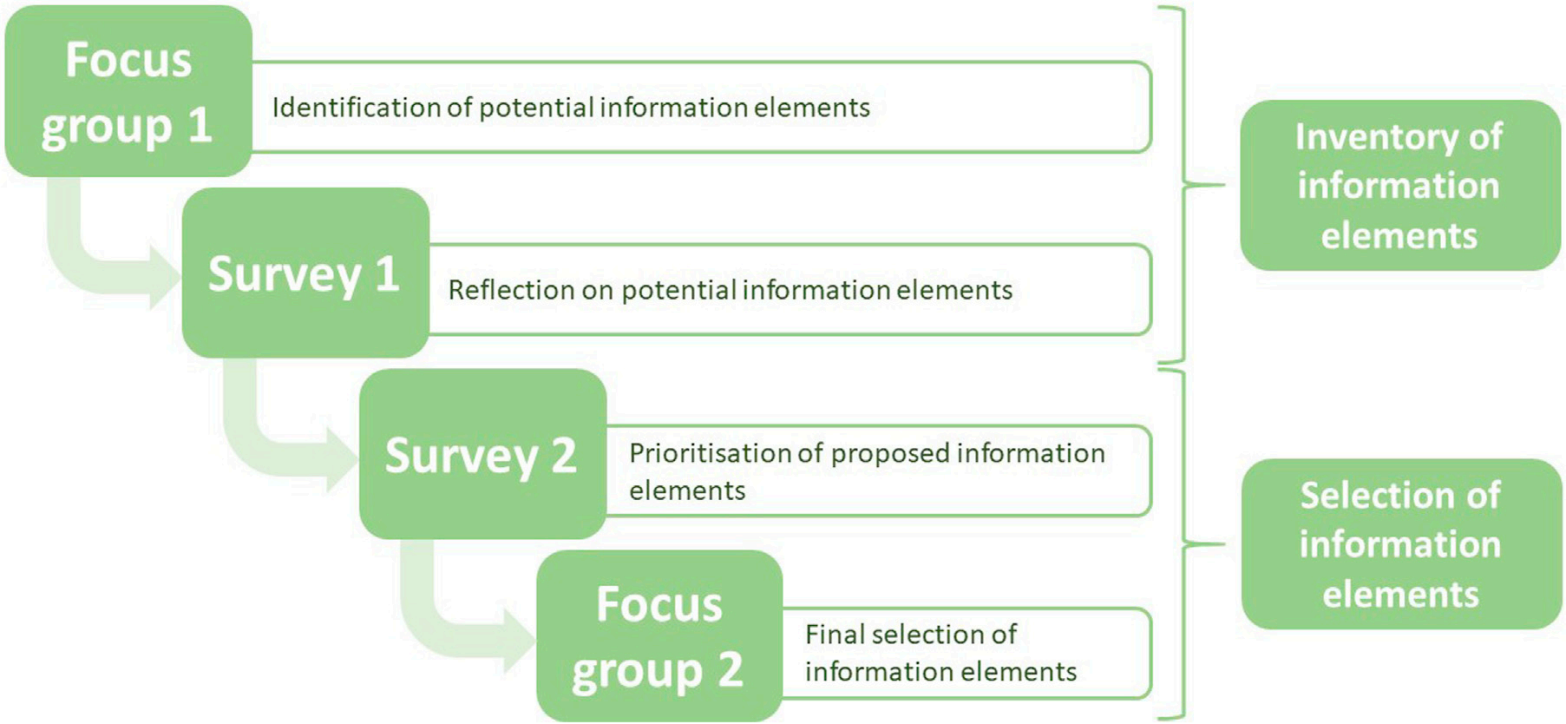
Flow-chart of study design in which elements were selected to assess the quality of information of case reports in pregnancy pharmacovigilance data.

This study aimed to identify elements that are necessary for the assessment of the quality of information for risk assessment of medicinal products used during pregnancy, following a sequential process. The process comprised four steps. In the first two steps, potential information needs were determined, by means of a focus group discussion and a survey based on the outcome of the focus group. This provided an overview of possible information elements to be selected. Steps three and four comprised a second survey and subsequent focus group discussion for a final selection of the elements (Figure 1). All steps are discussed in more detail below.

### 2.2 Inventory of potential elements of information

#### 2.2.1 Focus group 1: identification of potential information elements

The first step was to identify possible elements of information that are considered relevant when assessing the potential risk of exposure to a medicinal product during pregnancy. This first identification of information elements was done in a focus group with five pregnancy pharmacovigilance experts (two experts of the Dutch TIS and three scientists of Netherlands Pharmacovigilance Centre Lareb).

Since the information that is relevant for risk assessment may vary across different types of adverse events, a list of possible clinical scenarios was determined for which quality and causality assessment was considered relevant in case of medicinal product exposure during pregnancy. For example, a report concerning an early miscarriage requires different information compared to a report concerning a major congenital anomaly or a report concerning a pregnancy complication incurred by the mother.

With the proposed list of clinical scenarios in mind, a list of information elements that could be indicative of the clinical quality was created, taking into account the essential variables of the Core Data Elements (CDEs) as previously defined within the ConcePTION project. (ConcePTION, 2019; Richardson et al., 2023). These refer to variables that should ideally be collected in prospective reports regarding exposure to medicinal products during pregnancy based on existing coding systems, schemes and regulatory guidelines of reported medicinal product exposed pregnancies. (Richardson et al., 2023). Relevant elements of information for risk assessment might be a combination of information that is stored in a selection of variables as described in such coding systems, or might be stored in narratives only. Therefore, merely using the variables of the CDE for the purpose of this study would not suffice.

#### 2.2.2 Survey 1: reflection on potential information elements

A survey was created in which participants were asked to reflect on the proposed list of clinical scenarios and information elements provided, by means of open text questions (Supplementary Material S1). The first draft of the survey was evaluated by six pregnancy pharmacovigilance experts collaborating in Work Package 2 of the ConcePTION project or employed by Lareb for ambiguities and language errors. Participants were recruited via emails to members of Work Package 2 of the ConcePTION project and members of the Special Interest Group “women’s medicines” of the International Society of Pharmacovigilance (ISoP). In total 81 international experts were invited to participate. Responses were collected via the survey software LimeSurvey. (LimeSurvey GmbH Hamburg, 2017). A reminder was sent after 11 days, and the survey was closed after 15 days. Only completed responses were considered in the analyses.

### 2.3 Selection of elements of information

#### 2.3.1 Survey 2: prioritisation of proposed information elements

A second survey was created to determine which of the previously identified elements of information are considered to be most relevant for the risk assessment (Supplementary Material S2). The survey consisted of a number of multiple-choice questions in which participants were asked, per presented potential information element, to assess in which clinical scenario(s) the information of the element was important to be present in order to perform a reliable risk assessment. Both the clinical scenarios and the presented potential information elements were based on the results of survey 1. Before distribution, survey 2 was evaluated by the same experts who reviewed survey 1, for ambiguities and language errors. The group of experts contacted for the first survey was contacted once more via email to participate. Responses were collected via LimeSurvey. (LimeSurvey GmbH Hamburg, 2017). A reminder was sent after 7 days, and the survey was closed after 12 days. Only completed responses were taken into account.

Results of the second survey were discussed among the research team in order to decide which elements of information should be in- or excluded in the final selection. Per element, percentages of respondents that marked the element as relevant to a clinical scenario were calculated. If an element was marked as relevant in at least one clinical scenario by ≥ 75% of respondents, the element was included in the final list. If ≥ 40% of respondents did not consider the presented element relevant in any of the clinical scenarios presented, the element was excluded from the final list. Cut-offs were selected on subjective grounds.

#### 2.3.2 Focus group 2: final selection of information elements

In the first survey a question was included to recruit participants for the final focus group discussion. All participants who answered this question positively were invited for the teleconference. There, the complete list of potential elements of information as used in survey 2 was presented and the following situations discussed: 1) elements that could not be categorised as in- or excluded based on the criteria mentioned above; 2) elements that could possibly be merged; 3) elements about which the research team or participants of the focus group expressed any doubt regarding the direct in- or exclusion. Taking into account the comments made during the focus group discussion, the final list of information elements was created by the research team (YvRW, KC, EvP).

## 3 Results

Table 1 shows the demographics of the respondents to both surveys and the participants of the focus group discussion. Twenty-three experts responded to survey 1, the majority of whom were involved in the assessment of pregnancy data via TIS centres (*n* = 9) or the pharmaceutical industry (*n* = 9). The median number of years of experience in the analysis of pregnancy related information was 10.5 years (IQR 10 years). For survey 2, 18 experts responded, of whom 13 also participated in survey 1. Most were involved in the analysis of pregnancy related information via TIS centres (*n* = 10) (median time 12 years; IQR 6.5 years). In the focus group discussion seven experts participated, all of whom participated in survey 1 and six in survey 2. Most were involved in the assessment of pregnancy data via TIS centres (*n* = 3) or academia (*n* = 3), and the median number of years of experience with pregnancy data was 11 years (IQR 10 years).

**TABLE 1:** Respondents and participants of both surveys and both focus group discussions. PV, pharmacovigilance; TIS, Teratology Information Service; ICSRs, International Case Safety Reports; IQR, Interquartile Range.^a^, multiple options could apply.

		Focus group 1: identification of potential information elements	Survey 1: reflection on potential information elements	Survey 2: prioritisation of proposed information elements	Focus group 2: final selection of information elements
General	Complete responses	5	23	18	7
	Participants				
Previous response	Survey 1			13	7
	Survey 2				6
Profession (by training)^a^	Physician	2	5	3	0
	Pharmacist	1	6	6	2
	Teratologist	2	3	5	1
	Epidemiologist	2	4	4	2
	Pharmacologist	0	2	1	1
	Other research	1	6	6	2
	PV expert	1	3	0	2
Involved in assessment of pregnancy data via^a^	TIS	2	9	10	3
	PV centre	5	8	8	2
	Pregnancy registry	2	4	4	1
	Pharmaceutical industry	0	9	4	2
	Regulatory agency	0	2	3	2
	Academia	0	5	2	3
	Other	0	2	0	0
Type of data respondents worked with^a^	ICSRs	2	15	12	5
	Case reports in literature	3	12	9	3
	Pregnancy registries	2	17	12	4
	Counselling (TIS)	2	9	9	3
	Other	0	8	3	1
Years of experience with pregnancy data	Median	15	10.5	12	11
	IQR	10	10	6.5	10
	# Unknown	0	1	0	1

Exactly which elements are indicative of high quality of information (thereby supporting medical assessment of causality) varies with the type of adverse event. The applicability of the list of information elements as selected in this study is therefore based on the following clinical scenarios following the discussion in focus group 1: pregnancy loss, congenital anomalies and chromosomal or genetic defects, foetal complications, neonatal complications, complications of the infant or child, maternal pregnancy related complications, or no complications in pregnancy or the child.

Based on the results of the surveys and the focus group discussions, the final selection of 21 elements of information was made. These elements were grouped into seven categories, with 1-4 elements per category (Table 2).

**TABLE 2:** Elements needed to assess the quality of information for risk assessment of pregnancy pharmacovigilance data. The most right column shows a fictional example of a case report of small for gestational age (SGA) with the use of Product X during pregnancy.

Category	Elements of information	Example
Association	Medicinal product—event combination	Small for gestational age baby after maternal exposure to Product X
Event details	Information to validate the diagnosis of the event (e.g., test results)	1,356 g at 31+6 weeks gestation
	Timing of occurrence or detection of the event	Birth
	Chronologic evolution of the event, possibly in relation to the exposure	Normal weight 1 year after birth
Medicinal product exposure details	Administration information of medicinal product (e.g., dose, route)	10 mg tablet, once daily, oral
	Timing of exposure of medicinal product in relation to timing of gestation	Used from 6 months before LMP to week 10 of gestation
	Indication for use of medicinal product	Indication Z
	Other exposures (including additional information on e.g., dose, route, timing, and indication)	Folic acid 0.5 mg from 3 months before LMP to end of pregnancy; pertussis vaccination at 22 weeks’ gestation
Maternal factors	Medical history and concurrent disorders of the mother	History of seasonal allergies; concurrent ADHD
	Maternal demographics (e.g., age, weight)	Age at delivery 31; pre-pregnancy weight 72 kg; height 1.69 m
	Life style and risk factors (e.g., smoking, alcohol)	Never smoked; alcohol consumption until positive test at 5 weeks’ gestation (max 1 unit of alcohol per week); occupational exposure to low dose radiation
Pregnancy	Previous pregnancies	1 previous miscarriage at 8 weeks; 1 previous healthy child
	Pregnancy-related complications of current pregnancy	No complications
	Prenatal testing	Ultrasounds at 12 and 20 weeks showed normal growth (40th centile)
Labour	Labour onset	Spontaneous contractions
	Mode of delivery	Vaginal delivery
	Delivery complications	No complications
Child	Gestational age at birth	31 + 6
	Apgar score (1–5–10 min)	8/10/10
	Breastfeeding	Exclusively bottle fed
	Medical information of neonate (e.g., weight, diagnoses that are not the reported event)	Male; birth weight 1,356 g; eczema

## 4 Discussion

This study aimed to identify elements that are necessary to assess the quality of information of case reports in pregnancy pharmacovigilance data. Assessing the clinical quality of case reports in pregnancy pharmacovigilance data is important, because high quality data collection informs medical causality assessment, and leads to a more reliable and efficient signal detection process. (Meyboom et al., 1997; Oosterhuis et al., 2018). Additionally, assessment of the clinical quality of information of individual case reports can help improve reporting of suspected adverse reactions and publishing of case reports in literature.

Twenty-one elements of information were identified, divided into seven categories. The first category covers the association under study, which includes the medicinal product-event combination. This information will always need to be available when assessing for a possible causal association between exposure and outcome, therefore presence of this information will not distinguish between good or bad clinical quality of the information in a report. However, as the information is vital for the causality assessment, the element was included nonetheless.

The category “event details” contains elements of information that confirm the diagnosis (validation) and provide insight into time-related aspects (timing of occurrence or detection and evolution). This information is important to link to timing of exposure of the suspect product in order to reliably assess the risk, especially in case of disorders that are related to a specific gestational period, such as certain congenital anomalies. The category “medicinal product exposure details” contains elements that provide more details about how (administration information), when (timing of exposure), and why (indication) the mother was exposed to the suspect product. Additionally, the element ‘other exposures’ serves to rule out other causes of the clinical scenario, for example, inappropriate use of folic acid or exposure to known teratogens. In this element all relevant information regarding other exposures should be included, such as the route and dose of administration, timing of exposure in gestation and the indication for use.

The categories “maternal factors” and “pregnancy” provide insight into characteristics of the mother and pregnancy that could increase or decrease the chance of a causal relationship. Common factors that were included as information elements are the family medical history and concurrent disorders of the mother, maternal demographics, life style and risk factors, number and outcomes of previous pregnancies, pregnancy-related complications of the current pregnancy, and prenatal testing. Similarly, possible risk factors for the clinical scenario that are specifically related to the category “labour” or to the category “child” are included in the following information elements: labour onset, mode of delivery, delivery complications, gestational age at birth, Apgar score, breastfeeding, and medical information regarding the neonate.

Richardson et al. (2023), describe the process of the development of CDEs that are essential in pregnancy pharmacovigilance). This list contains variables that should be collected in prospective reports regarding exposure to medicinal products during pregnancy. The difference with the list established in this study is that CDEs are individual variables that are needed for optimal pregnancy data collection, while the elements as presented in Table 2 are sections of information that are needed to assess the risk. For example, the CDE contain individual variables such as the date of last menstrual period, the expected date of delivery, and the start and end date of medicinal product exposure. For risk assessment, the information element “timing of exposure” would summarize those individual variables into the information needed for risk assessment. It does not matter whether the information is directly reported (e.g., “exposure to product in from week 5–20 of gestation”) or whether the information can be derived from the individual variables (e.g., LMP and start and end date of exposure are reported). Table 2 shows a fictional example of a case where the information is divided into the information elements.

Additionally, the CDEs were developed from the perspective of existing data collection methods, while the set described in this study used the start point of practical situations such as the clinical scenarios that could be reported regarding medicinal product exposure during pregnancy. Similarly to the CDE, the draft EMA guideline on good pharmacovigilance practices for pregnancy specifically aims to provide guidance to MAHs and authorities for facilitating appropriate pharmacovigilance at aggregate data level, while the set in this study was developed for individual case analysis (European Medicines Agency, 2019).

### 4.1 Strengths and limitations

The group of pregnancy pharmacovigilance experts who participated in the selection of information elements in this study was multi-national, multi-disciplinary, and included practising clinicians, academics, pharmacists and pharmacologists (Table 1). The conclusions were drawn after a focus group discussion, two surveys, and another focus group discussion, hence we have confidence in the derived list of information elements. Additionally, the information elements were selected against the starting point of several predefined clinical scenarios relevant for medicinal product exposure related to pregnancy. This increases the likelihood that relevant elements were not overlooked.

Analysis of the answers supplied in the surveys showed that several experts did not fully understand the objective of the information elements at the time of responding to the questions in the surveys. It was difficult for respondents to distinguish between information needed in order to be able to perform a risk assessment and the actual risk assessment. The effect of these responses was possibly minimized by the discussion of the results in the focus group, where potential ambiguities were discussed, and clarified.

Additionally, in the second survey, the clinical scenario of foetal complications was inadvertently left out as an answer option for all but one element of information. This leads to a risk of elements being excluded from the list that should have been included. However, this limitation was mentioned in the focus group discussion, which then would have been able to counteract the effect of this absent element, if it were necessary.

## Data Availability

The raw data supporting the conclusion of this article will be made available by the authors, without undue reservation.

## 5 Conclusion

This study identified 21 elements deemed necessary for the assessment of quality of information of case reports in pregnancy pharmacovigilance data, via an extensive four-step process with the starting point of clinical scenarios. In future research this list will be reshaped into a tool to quantify the clinical quality of case reports in pregnancy pharmacovigilance data. The tool will be validated against expert opinion. Once validated, use of the tool for evaluating the clinical quality of reported pregnancy exposure data could help improve pregnancy pharmacovigilance data collection.
