# Supplementary material for: Elements to assess the quality of information of case reports in pregnancy pharmacovigilance data—a ConcePTION project
Source: Front Drug Saf Regul. 2023 Jun 29;3:1187888. doi: 10.3389/fdsfr.2023.1187888 (PMC12443074; doi:10.3389/fdsfr.2023.1187888)
Supplement: Supplementary file 1 [file Table1.DOCX]

Supplementary Material

Elements to Assess the Quality of Information of Case Reports in Pregnancy Pharmacovigilance Data - a ConcePTION project

**Yrea R. J. van Rijt-Weetink, Khoezik Chamani, Toine C. G. Egberts, Florence P. A. M. van Hunsel, David J. Lewis, Laura M. Yates, Ursula Winterfeld, Eugène P. van Puijenbroek***

*** Correspondence:** Eugène P. van Puijenbroek: e.vanpuijenbroek@lareb.nl

# Survey 1

**Development of a quality assessment tool for the use of medicines in pregnancy,**

**Part 1 of 2: Which elements are important when assessing the quality of safety data?**

Background

Information on the safety of the use of medicinal products during pregnancy is generally limited at the time the medicinal product is approved for marketing. Therefore, the clinical information regarding a suspected adverse event following use post-marketing serves as an important source of information.

Work Package 2 of the IMI funded ConcePTION project (www.imi-conception.eu) aims to improve the collection, analysis and interpretation of pregnancy pharmacovigilance data. **One of the areas to be addressed is the quality of information provided in reported cases in various available data sources for the assessment of the safety of medicinal product use during pregnancy**. For this, we focus on the quality of information described in the individual reports/cases. As a first step, a tool dedicated to measure the quality of these case reports, will be developed.

The objective of this study is to **create and validate a new tool (PregDoc) specifically optimized for the assessment of the quality of reported data related to exposures to medicines during pregnancy.** The tool should be suitable for use with all sources of pregnancy data, such as spontaneous reports, case reports published in literature, Teratology Information Service (TIS) data, registry data, and enhanced PV programme data.

Input required

**We would like to hear the thoughts of those working with primary data collections aimed at the detection of potential safety concerns for the use of medicinal products during pregnancy.**

Survey setup

We will be conducting two surveys to determine **which data elements are important when assessing the quality of pregnancy data**, and should therefore be included in PregDoc. **The main focus for our first survey is to find additional elements that could be of importance for the PregDoc tool.** Your suggestions will be incorporated into the second survey, of which the key focus will be on which variables are most relevant for assessing the safety of medicinal product use during pregnancy. The results of both surveys will be evaluated to inform the structure of the final PregDoc quality tool.

The survey will take approximately 15 to 30 minutes to complete. Your response is completely anonymous. If you have any questions about the survey or need technical support, please contact: y.weetink@lareb.nl

We appreciate your time, your participation and we welcome your feedback.

**Professional background:**

The questions on this page are about your experience/involvement with pregnancy data and your professional background.

What is your profession by training?

 Check all that apply

Community pharmacist

Hospital pharmacist

Epidemiologist

General practitioner

Gynaecologist

Paediatrician

Clinical geneticist

Teratologist

Other, please specify in the next question

Please specify here:

In which way(s) are you currently, or have you previously been, involved in the assessment of safety data related to pregnancy?

Check all that apply

Teratology Information Service

Pharmacovigilance Centre

Dedicated pregnancy registry

Pharmaceutical Industry

Regulatory agency (National or EMA)

Academia

Other, please specify in the next question

Please specify here:

What type of pregnancy data do you usually work with?

Check all that apply

Spontaneous data (Individual Case Safety Reports)

Case reports published in literature

Pregnancy registries

Counseling (TIS)

Other, please specify in the next question

Please specify here:

How long have you been involved in working with data related to medicinal product use during pregnancy? Please round your answer to the nearest year.

**Categories of pregnancy complications:**

When assessing cases on the safety of medicinal product use during pregnancy, the required information may vary per reported pregnancy complication or outcome. As an example, the data needed for the assessment of a case report regarding a spontaneous abortion differs from a report regarding a major malformation, or of a report regarding a long-term neurodevelopmental outcome. **Various symptoms and diagnoses therefore require different data to make a proper assessment. It is therefore essential to keep in mind what information is required to describe different complications and outcomes when assessing the quality of the available information reported for each individual case.**

The following question relates to the various categories of pregnancy complications or outcomes we may distinguish.

We pre-defined the following main categories of complications and outcomes that could be distinguished when assessing the safety of medicinal product use during pregnancy:

- Pregnancy Loss (stillbirth, induced termination, spontaneous abortion, ectopic pregnancy, molar pregnancy, blighted ovum)
- Congenital anomalies (minor, major, other) and chromosomal/genetic defects (e.g. Down’s syndrome, spinal muscular atrophy)
- Fetal complications/neo-natal complications (e.g. growth related complications, asphyxia)
- Infant/child complications (e.g. death of live born infant, (neuro)developmental outcomes, other long-term outcomes)
- Maternal pregnancy related complications (e.g. death, gestational diabetes, pre-eclampsia)
- No adverse outcomes/normal pregnancy and birth with a healthy mother and neonate(s)

Is there a category that you think should be included additionally, or do you have other comments regarding these categories? Please elaborate:

**Information required for quality assessment:**

The information required for the quality assessment will be the subject of the next set of questions. Please bear in mind the six main categories listed below:

- Pregnancy Loss (stillbirth, induced termination, spontaneous abortion, ectopic pregnancy, molar pregnancy, blighted ovum)
- Congenital anomalies (minor, major, other) and chromosomal/genetic defects (e.g. Down’s syndrome, spinal muscular atrophy)
- Fetal complications/neo-natal complications (e.g. growth related complications, asphyxia)
- Infant/child complications (e.g. death of live born infant, (neuro)developmental outcomes, other long-term outcomes)
- Maternal pregnancy related complications (e.g. death, gestational diabetes, pre-eclampsia)
- No adverse outcomes/normal pregnancy and birth with a healthy mother and neonate(s)

We pre-defined the following variables for the quality assessment. The goal of the questions is to determine whether or not any variables have been overlooked. If you recommend variables that don't fit into any of the categories, please report them at the last question on this page.

• Outcome related variables

• Exposure related variables

• Maternal variables

• Child related variables

• Pregnancy related variables

• Labour related variables

Note: case reports regarding outcomes and complications related to paternal medicinal product exposure or exposure through breast feeding are out of the scope of this tool.

As part of the category “outcome related variables” the following items have been defined:

• Clear description of outcome or complication

• Certainty of diagnosis: relevant tests, treatment, lab values, visual material, etc.

• Timing of occurrence/detection (e.g. age of child/period in pregnancy/during delivery)

Do you have any comments on the items specified above? For example, are there any additional items within this category that you think should be included, or do you think these items should be structured differently? If so, please elaborate.

As part of the category “exposure related variables” the following items have been defined:

This section should be evaluated for the medicinal product in question.

• Name of exposure

• Route of administration

• Dose and duration

• Period of exposure in relation to the stage of pregnancy/gestation

• Indication

Do you have any comments on the items specified above? For example, are there any additional items within this category that you think should be included, or do you think these items should be structured differently? If so, please elaborate.

As part of the category “maternal variables” the following items have been defined:

• Relevant medical history, comorbidities and risk factors

• Age/weight (pre-pregnancy)/height/BMI

- Other relevant exposures: comedication, supplements (e.g. folic acid, vitamins)
- Life style or other risk factors (e.g. alcohol/illicit drugs/smoking, diet, sports, recent travels, family history)

Do you have any comments on the items specified above? For example, are there any additional items within this category that you think should be included, or do you think these items should be structured differently? If so, please elaborate.

As part of the category “child related variables” the following items have been defined:

• Gestational age at birth

• Birth weight/length/head circumference

• Sex

• APGAR score

• Other relevant medical conditions (not mentioned in outcome category)

Do you have any comments on the items specified above? For example, are there any additional items within this category that you think should be included, or do you think these items should be structured differently? If so, please elaborate.

As part of the category “pregnancy related variables” the following items have been defined:

• Date of Last Menstrual Period (LMP) or Estimated Date of Birth (EDOB)

• Information on previous pregnancies (number and outcomes)

• Information on prenatal testing performed

• Plurality

• Assisted conception

Do you have any comments on the items specified above? For example, are there any additional items within this category that you think should be included, or do you think these items should be structured differently? If so, please elaborate.

As part of the category “labour related variables” the following items have been defined:

• Labour onset (induced, spontaneous)

• Mode of delivery

• Delivery complications

Do you have any comments on the items specified above? For example, are there any additional items within this category that you think should be included, or do you think these items should be structured differently? If so, please elaborate.

Apart from these categories, are there additional items that you would like to bring to our attention?

**Closing questions:**

Do you have any additional comments, questions, or concerns you would like to share?

May we contact you for additional information or clarification of your answers if necessary? Please leave your email address below. After the surveys have been analyzed, the email addresses will be deleted. **Yes/no with open text for email**

The PregDoc tool will be adjusted based on the results of both surveys. On June 1^st^ 2021, from 15:30h to 17:00h, we will finalize the first version of the PregDoc tool within a focus group discussion. If you would like to participate in this discussion, please leave your email address below. The email addresses will be removed after the meeting.

Thank you for your response. You will receive the second survey around the end of April via email. We hope you will also take the time to participate in the second survey and give us your valuable feedback.
