# Supplementary material for: Elements to assess the quality of information of case reports in pregnancy pharmacovigilance data—a ConcePTION project
Source: Front Drug Saf Regul. 2023 Jun 29;3:1187888. doi: 10.3389/fdsfr.2023.1187888 (PMC12443074; doi:10.3389/fdsfr.2023.1187888)
Supplement: Supplementary file 2 [file Table2.DOCX]

Supplementary Material

Elements to Assess the Quality of Information of Case Reports in Pregnancy Pharmacovigilance Data - a ConcePTION project

**Yrea R. J. van Rijt-Weetink, Khoezik Chamani, Toine C. G. Egberts, Florence P. A. M. van Hunsel, David J. Lewis, Laura M. Yates, Ursula Winterfeld, Eugène P. van Puijenbroek***

*** Correspondence:** Eugène P. van Puijenbroek: e.vanpuijenbroek@lareb.nl

# Survey 2

**Development of PregDoc: a quality assessment tool for the use of medicines in pregnancy**

**Part 2 of 2: Which variables are relevant for assessing the safety of drug use during pregnancy?**

Measuring the quality of reports

This study is aimed at developing a dedicated tool (PregDoc) for the assessment of quality of cases regarding the use of medicinal products during pregnancy. The quality of information depends on the presence or absence of information considered to be relevant for the assessment of the causal relationship between medicinal products and reported events.

Survey 2

Earlier in April we sent you the first of 2 surveys. The focus for our first survey was to assess elements of information that could be relevant for the PregDoc tool. We have incorporated your suggestions received in the first survey as far as possible.

**The** **key focus of this second survey is to determine which of these variables are most relevant for assessing the safety of medicinal product use during pregnancy**. The outcome of both surveys will be used in the final PregDoc quality tool.

Input required

**We would like to hear the thoughts of those working with primary data collections aimed at the detection of potential safety concerns for the use of medicinal products during pregnancy.**

The second survey will take approximately 15 to 30 minutes to complete. Your response is completely anonymous unless you provide us with your email for follow-up. If you have any questions about the survey or need technical support, please contact: y.weetink@lareb.nl

We appreciate your time and participation and we welcome your feedback.

**Professional background:**

The questions on this page are about your experience/involvement with pregnancy data and your professional background. We realise these questions were also part of the first survey. However, the data are processed anonymously, therefore we would like to ask you to answer these questions again.

Have you taken part in the first survey?

Yes/no answer

What is your profession by training?

 Check all that apply

Community pharmacist

Hospital pharmacist

Epidemiologist

General practitioner

Gynaecologist

Paediatrician

Clinical geneticist

Teratologist

Other, please specify in the next question

Please specify here:

In which way(s) are you currently, or have you previously been, involved in the assessment of safety data related to pregnancy?

Check all that apply

Teratology Information Service

Pharmacovigilance Centre

Dedicated pregnancy registry

Pharmaceutical Industry

Regulatory agency (National or EMA)

Academia

Other, please specify in the next question

Please specify here:

What type of pregnancy data do you usually work with?

Check all that apply

Spontaneous data (Individual Case Safety Reports)

Case reports published in literature

Pregnancy registries

Counseling (TIS)

Other, please specify in the next question

Please specify here:

How long have you been involved in working with data related to medicinal product use during pregnancy? Please round your answer to the nearest year.

**Information required for quality assessment**

After reviewing your suggestions in survey 1 we identified a number of variables that may be used in quality assessment. The aim of the following questions is to decide which variables are *most relevant* in determining the safety of medicinal product use during pregnancy. These variables will finally be included in the PregDoc tool. The variables are divided into the following categories:

• Outcome related variables

• Exposure related variables

• Maternal variables

• Child related variables

• Variables associated with current pregnancy

• Labour related variables

When assessing cases on the safety of medicinal product use during pregnancy, the required information may vary per reported pregnancy complication or outcome. As an example, the data needed for the assessment of a case report regarding a spontaneous abortion differs from a report regarding a major malformation, or of a report regarding a long-term neurodevelopmental outcome. **Various symptoms and diagnoses therefore require different data to make a proper assessment.**

For example, in the case of miscarriage, it is very relevant to know whether a woman has had previous miscarriages and if and when she used specific medicinal products. However, labour related variables are not of relevance. Conversely, when assessing a report on asphyxia, labour related variables are very relevant, while previous miscarriages are of less importance.

When variables are not relevant for the assessment of a specific report, they will not be taken into account in the calculation of the quality assessment.

We would like you to consider what information is required to assess the quality of information in individual cases, keeping in mind a number of situations for which the tool can be applied.

The following categories of pregnancy complications and outcomes **are situations for which the tool can be applied:**

- Pregnancy loss (e.g. (spontaneous) abortion, stillbirth)
- Congenital anomalies (minor, major)
- Chromosomal/genetic defects
- Fetal complications (e.g. growth related complications)
- Neonatal complications (e.g. asphyxia)
- Infant/child complications (e.g. death of live born infant, (neuro)developmental outcomes, other long-term outcomes)
- (Maternal) pregnancy related complications (e.g. pre-eclampsia, preterm birth)
- No adverse outcomes/normal pregnancy and birth with a healthy mother and neonate(s)

Note: case reports regarding outcomes and complications related to paternal medicinal product exposure or exposure through breast feeding are out of the scope of this tool.

**On this page the outcome related variables will be discussed.**

Based on the outcome of the first survey, the following elements have been selected in the category of outcome related variables. For each element, please select the categories for which you consider the information in this element to be relevant.

1. **Clarity of description of outcome or complication (either as free text or coded information, when needed supported by e.g. time of follow-up**, **physical examination or other ways of validation)**

*Select the categories mentioned below for which you consider this information to be relevant. Multiple options may be selected.*

- Pregnancy loss
- Chromosomal and genetic defects
- Other congenital anomalies
- Foetal complications
- Neonatal complications
- Complications of the infant and child (including developmental disorders)
- (Maternal) pregnancy related complications
- No adverse outcome (healthy mother and neonate)
- None of the above

1. **Information on diagnostic procedures (e.g. physical examination, relevant tests,  diagnostic imaging, pathology reports)**

*Select the categories mentioned below for which you consider this information to be relevant. Multiple options may be selected.*

- Pregnancy loss
- Chromosomal and genetic defects
- Other congenital anomalies
- Foetal complications
- Neonatal complications
- Complications of the infant and child (including developmental disorders)
- (Maternal) pregnancy related complications
- No adverse outcome (healthy mother and neonate)
- None of the above

1. **Timing of occurrence/detection (e.g. age of child/period in pregnancy/during delivery)**

*Select the categories mentioned below for which you consider this information to be relevant. Multiple options may be selected.*

- Pregnancy loss
- Chromosomal and genetic defects
- Other congenital anomalies
- Foetal complications
- Neonatal complications
- Complications of the infant and child (including developmental disorders)
- (Maternal) pregnancy related complications
- No adverse outcome (healthy mother and neonate)
- None of the above

1. **Chronology and evolution of the ADR**

*Select the categories mentioned below for which you consider this information to be relevant. Multiple options may be selected.*

- Pregnancy loss
- Chromosomal and genetic defects
- Other congenital anomalies
- Foetal complications
- Neonatal complications
- Complications of the infant and child (including developmental disorders)
- (Maternal) pregnancy related complications
- No adverse outcome (healthy mother and neonate)
- None of the above

1. **Evaluation of alternative causes of the reported events**

*Select the categories mentioned below for which you consider this information to be relevant. Multiple options may be selected.*

- Pregnancy loss
- Chromosomal and genetic defects
- Other congenital anomalies
- Foetal complications
- Neonatal complications
- Complications of the infant and child (including developmental disorders)
- (Maternal) pregnancy related complications
- No adverse outcome (healthy mother and neonate)
- None of the above

1. **Interpretation of the reporter**

*Select the categories mentioned below for which you consider this information to be relevant. Multiple options may be selected.*

- Pregnancy loss
- Chromosomal and genetic defects
- Other congenital anomalies
- Foetal complications
- Neonatal complications
- Complications of the infant and child (including developmental disorders)
- (Maternal) pregnancy related complications
- No adverse outcome (healthy mother and neonate)
- None of the above

**On this page the exposure related variables will be discussed.**

Based on the outcome of the first survey, the following elements have been selected in the category of exposure related variables. For each element, please select the categories for which you consider the information in this element to be relevant.

1. **Exposure to suspected medicinal product**

*Select the categories mentioned below for which you consider this information to be relevant. Multiple options may be selected.*

- Pregnancy loss
- Chromosomal and genetic defects
- Other congenital anomalies
- Foetal complications
- Neonatal complications
- Complications of the infant and child (including developmental disorders)
- (Maternal) pregnancy related complications
- No adverse outcome (healthy mother and neonate)
- None of the above

1. **Route of administration**

*Select the categories mentioned below for which you consider this information to be relevant. Multiple options may be selected.*

- Pregnancy loss
- Chromosomal and genetic defects
- Other congenital anomalies
- Foetal complications
- Neonatal complications
- Complications of the infant and child (including developmental disorders)
- (Maternal) pregnancy related complications
- No adverse outcome (healthy mother and neonate)
- None of the above

1. **Daily dosage and duration**

*Select the categories mentioned below for which you consider this information to be relevant. Multiple options may be selected.*

- Pregnancy loss
- Chromosomal and genetic defects
- Other congenital anomalies
- Foetal complications
- Neonatal complications
- Complications of the infant and child (including developmental disorders)
- (Maternal) pregnancy related complications
- No adverse outcome (healthy mother and neonate)
- None of the above

1. **Timing of exposure in relation to the stage of pregnancy/gestation/LMP**

*Select the categories mentioned below for which you consider this information to be relevant. Multiple options may be selected.*

- Pregnancy loss
- Chromosomal and genetic defects
- Other congenital anomalies
- Foetal complications
- Neonatal complications
- Complications of the infant and child (including developmental disorders)
- (Maternal) pregnancy related complications
- No adverse outcome (healthy mother and neonate)
- None of the above

1. **Indication for use**

*Select the categories mentioned below for which you consider this information to be relevant. Multiple options may be selected.*

- Pregnancy loss
- Chromosomal and genetic defects
- Other congenital anomalies
- Foetal complications
- Neonatal complications
- Complications of the infant and child (including developmental disorders)
- (Maternal) pregnancy related complications
- No adverse outcome (healthy mother and neonate)
- None of the above

1. **Certainty of actual exposure to suspected medicinal product**

*Select the categories mentioned below for which you consider this information to be relevant. Multiple options may be selected.*

- Pregnancy loss
- Chromosomal and genetic defects
- Other congenital anomalies
- Foetal complications
- Neonatal complications
- Complications of the infant and child (including developmental disorders)
- (Maternal) pregnancy related complications
- No adverse outcome (healthy mother and neonate)
- None of the above

1. **Other relevant exposures (e.g. comedication, folic acid, supplements)**

*Select the categories mentioned below for which you consider this information to be relevant. Multiple options may be selected.*

- Pregnancy loss
- Chromosomal and genetic defects
- Other congenital anomalies
- Foetal complications
- Neonatal complications
- Complications of the infant and child (including developmental disorders)
- (Maternal) pregnancy related complications
- No adverse outcome (healthy mother and neonate)
- None of the above

1. **Relevant paternal preconceptional exposures**

*Select the categories mentioned below for which you consider this information to be relevant. Multiple options may be selected.*

- Pregnancy loss
- Chromosomal and genetic defects
- Other congenital anomalies
- Foetal complications
- Neonatal complications
- Complications of the infant and child (including developmental disorders)
- (Maternal) pregnancy related complications
- No adverse outcome (healthy mother and neonate)
- None of the above

**On this page the maternal variables will be discussed.**

Based on the outcome of the first survey, the following elements have been selected in the category of maternal variables. For each element, please select the categories for which you consider the information in this element to be relevant.

1. **Relevant medical and family/paternal history (e.g. anomalies, hereditary disorders, morbidities)**

*Select the categories mentioned below for which you consider this information to be relevant. Multiple options may be selected.*

- Pregnancy loss
- Chromosomal and genetic defects
- Other congenital anomalies
- Foetal complications
- Neonatal complications
- Complications of the infant and child (including developmental disorders)
- (Maternal) pregnancy related complications
- No adverse outcome (healthy mother and neonate)
- None of the above

1. **Maternal characteristics (e.g. age/weight (pre-pregnancy)/height/BMI)**

*Select the categories mentioned below for which you consider this information to be relevant. Multiple options may be selected.*

- Pregnancy loss
- Chromosomal and genetic defects
- Other congenital anomalies
- Foetal complications
- Neonatal complications
- Complications of the infant and child (including developmental disorders)
- (Maternal) pregnancy related complications
- No adverse outcome (healthy mother and neonate)
- None of the above

1. **Life style or other risk factors (e.g. alcohol/illicit drugs/smoking, diet, sports, recent travels, radiology exposure etc.)**

*Select the categories mentioned below for which you consider this information to be relevant. Multiple options may be selected.*

- Pregnancy loss
- Chromosomal and genetic defects
- Other congenital anomalies
- Foetal complications
- Neonatal complications
- Complications of the infant and child (including developmental disorders)
- (Maternal) pregnancy related complications
- No adverse outcome (healthy mother and neonate)
- None of the above

1. **Socioeconomic status (e.g.: education level, marital status, occupation, postal code)**

*Select the categories mentioned below for which you consider this information to be relevant. Multiple options may be selected.*

- Pregnancy loss
- Chromosomal and genetic defects
- Other congenital anomalies
- Foetal complications
- Neonatal complications
- Complications of the infant and child (including developmental disorders)
- (Maternal) pregnancy related complications
- No adverse outcome (healthy mother and neonate)
- None of the above

1. **Ethnicity**

*Select the categories mentioned below for which you consider this information to be relevant. Multiple options may be selected.*

- Pregnancy loss
- Chromosomal and genetic defects
- Other congenital anomalies
- Foetal complications
- Neonatal complications
- Complications of the infant and child (including developmental disorders)
- (Maternal) pregnancy related complications
- No adverse outcome (healthy mother and neonate)
- None of the above

1. **Information on previous pregnancies (number and outcomes)**

*Select the categories mentioned below for which you consider this information to be relevant. Multiple options may be selected.*

- Pregnancy loss
- Chromosomal and genetic defects
- Other congenital anomalies
- Foetal complications
- Neonatal complications
- Complications of the infant and child (including developmental disorders)
- (Maternal) pregnancy related complications
- No adverse outcome (healthy mother and neonate)
- None of the above

1. **Pregnancy related complications (e.g. pre-eclampsia, maternal infections, diabetes)**

*Select the categories mentioned below for which you consider this information to be relevant. Multiple options may be selected.*

- Pregnancy loss
- Chromosomal and genetic defects
- Other congenital anomalies
- Foetal complications
- Neonatal complications
- Complications of the infant and child (including developmental disorders)
- (Maternal) pregnancy related complications
- No adverse outcome (healthy mother and neonate)
- None of the above

**On this page the child related variables will be discussed.**

Based on the outcome of the first survey, the following elements have been selected in the category of child related variables. For each element, please select the categories for which you consider the information in this element to be relevant.

1. **Gestational age at birth/birth weight/length/head circumference**

*Select the categories mentioned below for which you consider this information to be relevant. Multiple options may be selected.*

- Pregnancy loss
- Chromosomal and genetic defects
- Other congenital anomalies
- Foetal complications
- Neonatal complications
- Complications of the infant and child (including developmental disorders)
- (Maternal) pregnancy related complications
- No adverse outcome (healthy mother and neonate)
- None of the above

1. **Date of birth**

*Select the categories mentioned below for which you consider this information to be relevant. Multiple options may be selected.*

- Pregnancy loss
- Chromosomal and genetic defects
- Other congenital anomalies
- Foetal complications
- Neonatal complications
- Complications of the infant and child (including developmental disorders)
- (Maternal) pregnancy related complications
- No adverse outcome (healthy mother and neonate)
- None of the above

1. **Sex**

*Select the categories mentioned below for which you consider this information to be relevant. Multiple options may be selected.*

- Pregnancy loss
- Chromosomal and genetic defects
- Other congenital anomalies
- Foetal complications
- Neonatal complications
- Complications of the infant and child (including developmental disorders)
- (Maternal) pregnancy related complications
- No adverse outcome (healthy mother and neonate)
- None of the above

1. **APGAR score**

*Select the categories mentioned below for which you consider this information to be relevant. Multiple options may be selected.*

- Pregnancy loss
- Chromosomal and genetic defects
- Other congenital anomalies
- Foetal complications
- Neonatal complications
- Complications of the infant and child (including developmental disorders)
- (Maternal) pregnancy related complications
- No adverse outcome (healthy mother and neonate)
- None of the above

1. **Information on breastfeeding?**

*Select the categories mentioned below for which you consider this information to be relevant. Multiple options may be selected.*

- Pregnancy loss
- Chromosomal and genetic defects
- Other congenital anomalies
- Foetal complications
- Neonatal complications
- Complications of the infant and child (including developmental disorders)
- (Maternal) pregnancy related complications
- No adverse outcome (healthy mother and neonate)
- None of the above

1. **Other relevant medical/neonatal topics after birth not part of outcome (e.g.: plurality, complications)**

*Select the categories mentioned below for which you consider this information to be relevant. Multiple options may be selected.*

- Pregnancy loss
- Chromosomal and genetic defects
- Other congenital anomalies
- Foetal complications
- Neonatal complications
- Complications of the infant and child (including developmental disorders)
- (Maternal) pregnancy related complications
- No adverse outcome (healthy mother and neonate)
- None of the above

**On this page the variables associated with the current pregnancy will be discussed.**

Based on the outcome of the first survey, the following elements have been selected in the category of variables related to the current pregnancy. For each element, please select the categories for which you consider the information in this element to be relevant.

1. **Date of Last Menstrual Period (LMP) or Estimated Date of Birth (EDOB)**

*Select the categories mentioned below for which you consider this information to be relevant. Multiple options may be selected.*

- Pregnancy loss
- Chromosomal and genetic defects
- Other congenital anomalies
- Foetal complications
- Neonatal complications
- Complications of the infant and child (including developmental disorders)
- (Maternal) pregnancy related complications
- No adverse outcome (healthy mother and neonate)
- None of the above

1. **Information on routine prenatal testing performed (e.g. pregnancy test, ultrasound)**

*Select the categories mentioned below for which you consider this information to be relevant. Multiple options may be selected.*

- Pregnancy loss
- Chromosomal and genetic defects
- Other congenital anomalies
- Foetal complications
- Neonatal complications
- Complications of the infant and child (including developmental disorders)
- (Maternal) pregnancy related complications
- No adverse outcome (healthy mother and neonate)
- None of the above

1. **Assisted conception**

*Select the categories mentioned below for which you consider this information to be relevant. Multiple options may be selected.*

- Pregnancy loss
- Chromosomal and genetic defects
- Other congenital anomalies
- Foetal complications
- Neonatal complications
- Complications of the infant and child (including developmental disorders)
- (Maternal) pregnancy related complications
- No adverse outcome (healthy mother and neonate)
- None of the above

**On this page the labour related variables will be discussed.**

Based on the outcome of the first survey, the following elements have been selected in the category of labour related variables. For each element, please select the categories for which you consider the information in this element to be relevant.

1. **Labour onset (induced, spontaneous)**

*Select the categories mentioned below for which you consider this information to be relevant. Multiple options may be selected.*

- Pregnancy loss
- Chromosomal and genetic defects
- Other congenital anomalies
- Foetal complications
- Neonatal complications
- Complications of the infant and child (including developmental disorders)
- (Maternal) pregnancy related complications
- No adverse outcome (healthy mother and neonate)
- None of the above

1. **Mode of delivery**

*Select the categories mentioned below for which you consider this information to be relevant. Multiple options may be selected.*

- Pregnancy loss
- Chromosomal and genetic defects
- Other congenital anomalies
- Foetal complications
- Neonatal complications
- Complications of the infant and child (including developmental disorders)
- (Maternal) pregnancy related complications
- No adverse outcome (healthy mother and neonate)
- None of the above

1. **Delivery complications (e.g. placenta previa, face/breech presentation)**

*Select the categories mentioned below for which you consider this information to be relevant. Multiple options may be selected.*

- Pregnancy loss
- Chromosomal and genetic defects
- Other congenital anomalies
- Foetal complications
- Neonatal complications
- Complications of the infant and child (including developmental disorders)
- (Maternal) pregnancy related complications
- No adverse outcome (healthy mother and neonate)
- None of the above

**Closing questions:**

Do you have any additional comments, questions, or concerns you would like to share?

May we contact you for additional information or clarification of your answers if necessary? Please leave your email address below. After the surveys have been analysed, the email addresses will be deleted.

Yes/no

If yes, please enter your email address here:

In June and July we will validate and test the PregDoc tool by comparing the quality of case reports assessed by use of the tool to the quality of case reports assessed by expert opinion. Would you be willing to use your expertise to assess the quality of approximately 30 cases (poor quality, moderate quality, good quality)? If you are happy for use to contact you for the expert assessment please leave your email address below. The email addresses will be deleted after the validation and testing of PregDoc.

Yes/no

If yes, please enter your email address here:

Thank you for taking the time to complete this survey. Your feedback will assist us in optimizing and improving the PregDoc tool.
